# Effects of photodynamic therapy on dermal fibroblasts from xeroderma pigmentosum and Gorlin-Goltz syndrome patients

**DOI:** 10.18632/oncotarget.20485

**Published:** 2017-08-24

**Authors:** Alicia Zamarrón, Marta García, Marcela Del Río, Fernando Larcher, Ángeles Juarranz

**Affiliations:** ^1^ Department of Biology, Faculty of Sciences, Autónoma University of Madrid, IRYCIS, Madrid, Spain; ^2^ Department of Bioengineering, Carlos III University (UC3M), Madrid, Spain; ^3^ CIEMAT-Centro de Investigaciones Biomédicas en Red de Enfermedades Raras (CIBERER), Madrid, Spain; ^4^ Instituto de Investigación Sanitaria de la Fundación Jiménez Díaz (IIS-FJD), Madrid, Spain

**Keywords:** photodynamic therapy, ultraviolet light, cancer-associated fibroblasts, Gorlin-Goltz syndrome, xeroderma pigmentosum

## Abstract

PDT is widely applied for the treatment of non-melanoma skin cancer pre-malignant and malignant lesions (actinic keratosis, basal cell carcinoma and *in situ* squamous cell carcinoma). In photodynamic therapy (PDT) the interaction of a photosensitizer (PS), light and oxygen leads to the formation of reactive oxygen species (ROS) and thus the selective tumor cells eradication. Xeroderma pigmentosum (XP) and Gorlin-Goltz Syndrome (GS) patients are at high risk of developing skin cancer in sun-exposed areas. Therefore, the use of PDT as a preventive treatment may constitute a very promising therapeutic modality for these syndromes. Given the demonstrated role of cancer associated fibroblasts (CAFs) in tumor progression and the putative CAFs features of some cancer-prone genodermatoses fibroblasts, in this study, we have further characterized the phenotype of XP and GS dermal fibroblasts and evaluated their response to methyl-δ-aminolevulinic acid (MAL)-PDT compared to that of dermal fibroblasts obtained from healthy donors. We show here that XP/GS fibroblasts display clear features of CAFs and present a significantly higher response to PDT, even after being stimulated with UV light, underscoring the value of this therapeutic approach for these rare skin conditions and likely to other forms of skin cancer were CAFs play a major role.

## INTRODUCTION

Ultraviolet radiation (UVR) from sun is the major etiologic factor responsible for skin cancer. Apart from the local immunosuppressive effects, UVA and UVB can induce DNA damage directly or through reactive oxygen species (ROS) production. Damaged cells, if not correctly repaired or eliminated, may survive with genetic alterations, causing aging and cancer [1–3]. Skin overexposure to UVR results in clearly mutagenic DNA lesions, being pyrimidine photoproducts such as cyclobutane pyrimidine dimers (CPDs) and pyrimidine (6-4) pyrimidone photoproducts (6-4PPs) the predominant forms of DNA adducts [3, 4].

The harmful effects caused by UVR overexposure on human skin are particularly relevant in patients with specific genetic disorders which lead to UV-light sensitivity. This is the case of Xeroderma pigmentosum (XP) and Gorlin-Goltz syndrome patients. Xeroderma pigmentosum (XP) is a rare, autosomal, recessive, genetic disorder that occurs as a result of mutations in genes involved in the nucleotide excision repair (NER) pathway, making defective the responses of XP cells to photoproducts induced in DNA by UVR from sunlight. Patients with this syndrome have a very high predisposition for developing skin cancers in sun-exposed areas due to this diminished DNA repair activity [2, 5, 6]. XP patients develop both non-melanocytic cancer (squamous cell carcinomas, SCCs; and basal cells carcinomas, BCCs) and malignant melanoma with a frequency higher than 10000 and 2000 fold respectively than in the general population [6]. Gorlin-Goltz syndrome (GS), also known as nevoid basal cell carcinoma syndrome (NBCCS), is a genetic disorder inherited in a dominant autosomal way whose patients are predisposing to develop multiple (from several to thousands) BCCs. Mutations in genes of Hedgehog pathway has been identified as the cause of this syndrome. Most mutations occur in Patched (PTCH 1) gene, although can also occur in Smoothened (SMO) gene [7–10].

A strict protection regimen from sunlight combined with conventional surgery or cryotherapy is the mainstay of traditional treatment of these syndromes. However, because of 90% of lesions are located in sun-exposed sites such as face or neck, it can result in a significant disfigurement [6, 2, 11]. Moreover, in many cases, the large number of lesions in the same area makes traditional therapeutic modalities impractical [12].

In this sense, photodynamic therapy (PDT), a non-invasive modality widely used in dermatology mainly for the treatment of some variants of non-melanoma skin cancer (NMSC), has the advantage of achieving a good cosmetic outcome. PDT is based on the interaction of a photosensitizer (PS), light and oxygen, that leads to the formation of ROS and thus the eradication of treated lesion [13, 14]. ROS are extremely harmful for cell viability. Their intracellular accumulation cause the oxidation and subsequent functional inactivation of several cell components and can also severely disturbing DNA replication and transcription mechanisms. All these processes can activate irreversible apoptotic and necrotic cell death mechanisms and the induced oxidative stress is associated with several human diseases [15, 16].

The possibility of using PDT as a preventive treatment for XP and GS syndromes constitutes a very interesting research area. Larson and Cunninghan [17] reported good results applying PDT to affected areas in a patient with XP. Furthermore, clinical studies carried out by Segura et al. [18] showed that topical PDT with MAL and red light may be useful for the treatment of superficial BCC in GS and XP patients.

There are many evidences indicating that tumor microenvironment plays a prominent role in the onset, growth, and proliferation of neoplastic cells. Cancer cells can activate their stroma and trigger the remodeling of extracellular matrix (ECM) promoting tumor growth. Moreover, the tumor microenvironment can also generate oxidative damage and genetic instability, stimulating invasive capacity of cancer cells [19–21]. In this context, dermal fibroblasts obtained from skin biopsies of patients with XP or GS syndromes would be a key determinant in investigating factors involved in the malignant progression and spread of skin cancer and could represent an important target for novel cancer therapies.

This study mainly focused on evaluating the effects of MAL-PDT on primary dermal fibroblasts from XP and GS patients. We have analyzed the possible differences in their responses to the treatment and then have compared them to the response in healthy fibroblasts. Moreover, we have also assessed whether the effects of MAL-PDT were sustained when these fibroblasts were stimulated with UVA and UVB light at sub-genotoxic doses. It can be expected that XP and GS fibroblasts show characteristics of cancer-associated fibroblasts (CAFs), playing an important role in several aspects of the tumor progression, promoting cancer cell growth, invasiveness and angiogenesis [19, 22, 23], so they could be excellent potential targets for PDT to prevent the appearance of skin cancer in XP and GS patients.

## RESULTS

### Characterization of XP and GS fibroblasts: analysis of CAFs markers

**Figure 1:**
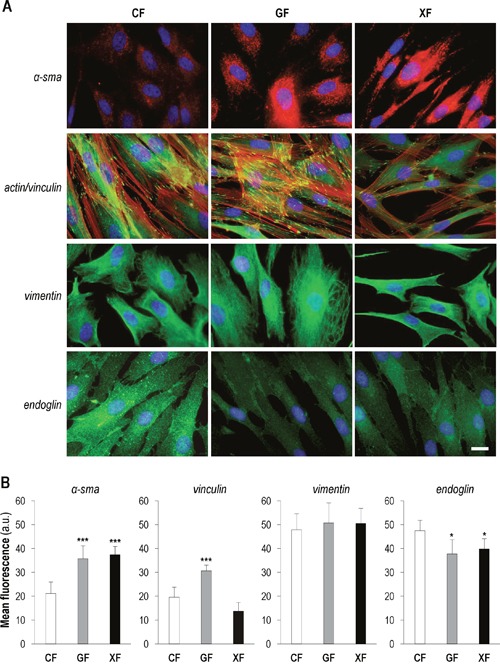
Expression of CAF markers in primary dermal fibroblasts **(A)** Immunofluorescence detection of α-sma, vinculin/F-actin, vimentin and endoglin in *CF*, *GF* and *XF* (scale bar: 10 μm). **(B)** Mean intensity of the fluorescent signal of each marker, measured by using ImageJ (total quantified area: 4 cm^2^) (*: significant *P*<0.1; **: significant *P*<0.05; ***: significant *P*<0.01).

To characterize the putative CAF features of fibroblasts obtained from XP and GS donors, we evaluated the protein expression pattern (Figure 1A) and the intensity of the fluorescent signal (Figure 1B) of α-sma, vinculin/F-actin, vimentin and endoglin by immunofluorescence. Analysis of α-sma revealed a significantly stronger signal in *GF* and *XF* compared to *CF* (*P*<0.01). Differences were also evident regarding α-sma expression pattern: well organized and arranged in visible fibers in *XF* and *GF*, while diffused in the cytoplasm in *CF*. Fluorescent signal of vinculin, a protein involved in the regulation of focal adhesions (FAs), was significantly more intense in *GF* than in *XF* and *CF* (*P*<0.01), suggesting the presence of a higher number of focal adhesion contacts in *GF*. Moreover, the counterstaining with F-actin also revealed a higher number of stress fibers in *GF* than in *XF* and *CF*. No differences on signal intensity in the three cell types for the mesenchymal marker vimentin were found. However, vimentin intermediate filaments displayed higher level of organization in *GF*. Finally, given its important role in TGFβ signaling, we also analyzed the expression of the cell-surface glycoprotein endoglin (or CD105), noting that its expression was significantly lower in *GF* and *XF* as compared with *CF* (*P*<0.1).

### Effects of MAL-PDT in fibroblasts survival

**Figure 2:**
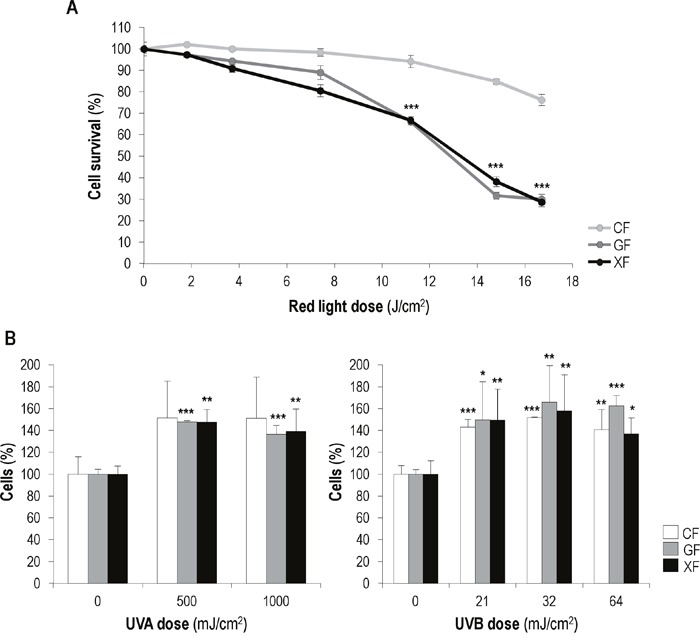
Effects of MAL-PDT and UV irradiation on fibroblasts survival **(A)** *CF*, *GF* and *XF* were incubated with 1 mM of MAL for 5 h and then subjected to different red light doses (ranging from 0 to 17 J/cm^2^). Cell survival was assessed 24 h after PDT by the trypan blue dye exclusion assay and counting by using an Automated Cell Counter. **(B)** Percentages of fibroblasts present in the cultures 48 h after UVA (500 and 1000 mJ/cm^2^) and UVB (21, 32 and 64 mJ/cm^2^) irradiation. Graphs show the significant differences between irradiated cultures and their corresponding non-irradiated (control) cultures (*: significant *P*<0.1; **: significant *P*<0.05; ***: significant *P*<0.01).

To evaluate the effect of MAL-based PDT on the three fibroblasts types, cells were incubated with MAL (1mM) for 5 h and then exposed to different red light doses (from 1.8 to 16.7 J/cm^2^) (Figure 2A). PDT induced a cytotoxic effect on all cell types which was dependent on the red light dose, but at light doses equal or higher than 11.2 J/cm^2^ *GF* and *XF* were more sensitive to the treatment than *CF* (*P*<0.01). Treatment conditions that caused a 33,8±1,4% of cell death in *GF* and a 33,2±1,6% *XF* (1 mM of MAL plus 11.2 J/cm^2^ of red light), induced a 5,6±2,8% in *CF*.

PDT effects were also evident on cell morphology (Supplementary Figure 2A). In fact phase contrast microscope images showed that PDT caused cytoplasmic retraction and cellular stretching, indicative of cell degeneration processes that were more apparent in *GF* and *XF*.

### Effects of UVA and UVB low doses in fibroblasts proliferation and morphology

The effect of UVA and UVB light on the proliferation rate of *CF*, *GF* and *XF* cells was assessed by cell counting 24, 48 and 72 h after irradiation. In general, both UVA and UVB applied doses led to a slight increase in fibroblasts proliferation, which was more marked 48 h after irradiation. At this time point (Figure 2B) in the case of UVA exposure no significant differences were observed between irradiated and non-irradiated *CF* cell percentage, while *GF* and *XF* irradiated cultures showed a significantly higher cell percentage than non-irradiated ones. Exposure to UVB light resulted in a significant increase of cell percentage in the three fibroblast types. In any case, the UVA and UVB low doses used did not caused apparent changes in cellular morphology (Supplementary Figure 2B).

### Analysis of DNA damage markers after UVA/UVB exposure

**Figure 3:**
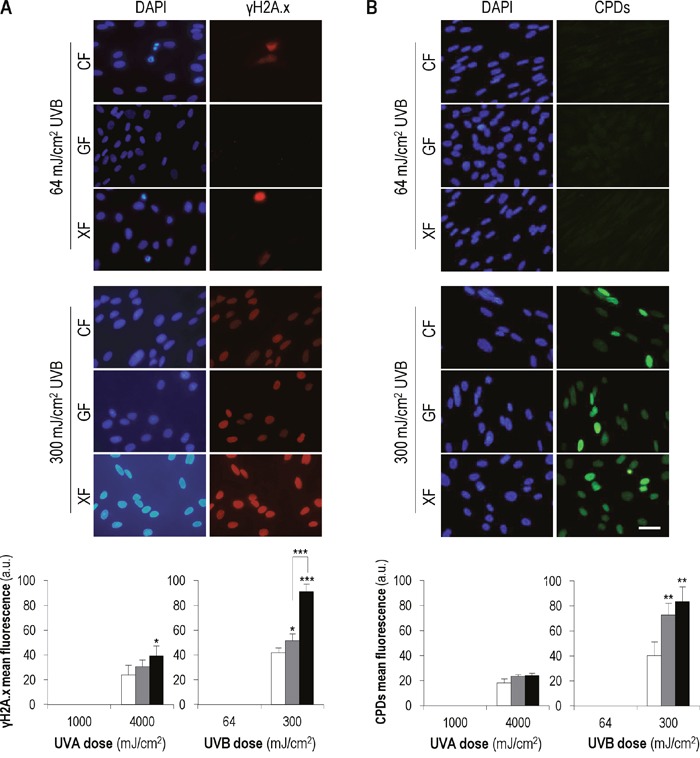
γH2A.x expression and CPDs formation after UVA and UVB irradiation Cell cultures were sunjected to UVA/UVB low doses (1000 and 64 mJ/cm^2^, respectively) and UVA/UVB high doses (4000 and 300 mJ/cm^2^, respectively). **(A)** The signal of γH2A.x and the CPDs foci were detected by immunofluorescence. Images correspond to UVB-exposed cultures. Similar patterns were observed after UVA irradiation (data not shown). **(B)** Mean intensity of the fluorescent signals of γH2A.x and CPDs was measured using the Measure and Label plugin of ImageJ software (*: significant *P*<0.1; **: significant *P*<0.05; ***: significant *P*<0.01) (MF: mean fluorescence).

To confirm that UVA and UVB at the doses used did not induce DNA damage, we analyzed the nuclear expression of γH2A.x, the active/phosphorylated form of the histone H2A.X, and the formation of cyclobutane pyrimidine dimers (CPDs), dipyrimidine photoproducts induced by UV radiation, by immunofluorescence (Figure 3). Measurement of both markers was carried out 48 h after UVA (1000 mJ/cm^2^) and UVB (64 mJ/cm^2^) exposure. These light doses did not induce γH2A.x expression in interphase nuclei and the signal of this protein was only observed in nuclei of dividing cells. Similarly, no CPD foci were detected in nuclei of cells subjected to UV low doses. However, when *CF*, *GF* and *XF* were subjected to higher UVA and UVB doses (4000 and 300 mJ/cm^2^, respectively), used as positive control of damage, γH2A.x signal as well as CPDs foci were observed in all nuclei, confirming DNA damage. Moreover, in the case of UVB high dose, the mean fluorescence of both markers was significantly higher in *GF* and *XF* with respect to *CF*. Fluorescence images correspond to UVB-exposed cultures. The same expression patterns were observed after UVA exposure (data not shown).

Overall, our γH2A.x and CPDs results indicate that light doses equal or lower than 1000 mJ/cm^2^ of UVA and 64 mJ/cm^2^ of UVB, do not induce any detectable DNA repair-triggering damage on *CF*, *GF* and *XF* fibroblasts.

### Production of reactive oxygen species (ROS) after UVA exposure and PDT

**Figure 4:**
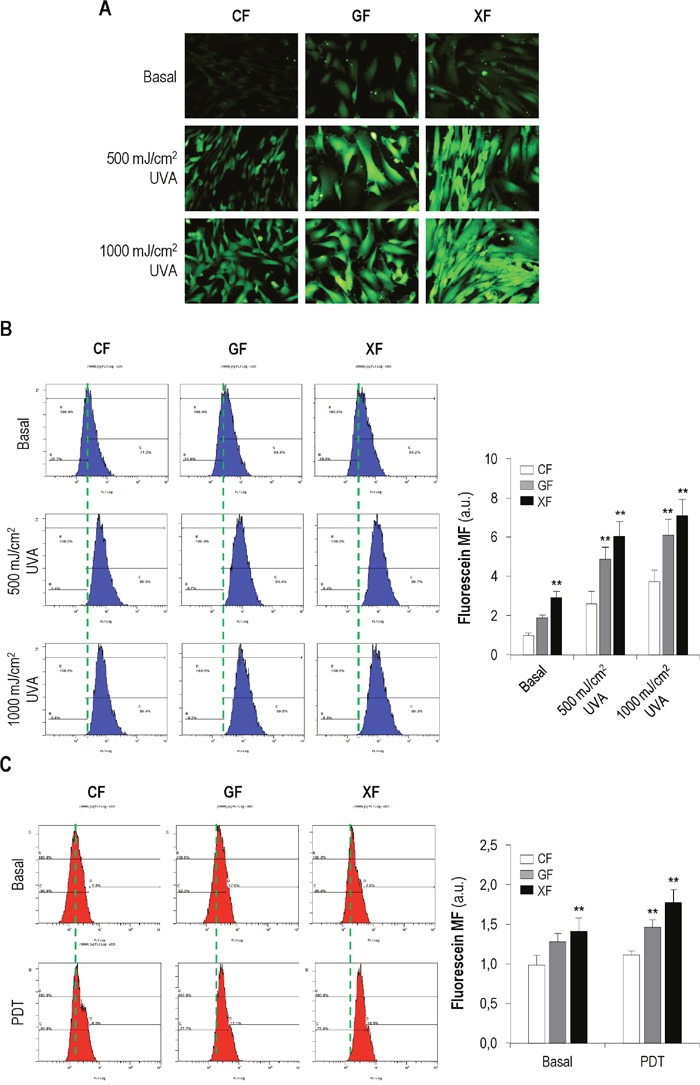
Production of ROS after UVA light **(A-B)** and PDT **(C)**. (A-B) Cell cultures were incubated with DHF-DA and exposed to UVA light (500 or 1000 mJ/cm^2^). Then, the fluorescent signal corresponding to fluorescein was observed using a fluorescence microscopy (A) and measured by flow cytometry (B) (*P*<0.05). (C) Cells were subjected to PDT (MAL 1mM plus 11.2 J/cm^2^) and the fluorescence of fluorescein was measured by flow cytometry (*P*<0.05). (Cells incubated with DHF-DA but not exposed to UVA light were used as control, baseline fluorescein).

UV effects can be mediated by the production of reactive oxygen species (ROS). In the same way, ROS are the main cytotoxic agents triggered by PDT. Therefore, we analyzed the intracellular ROS concentration in *CF*, *GF* and *XF* when subjected to UVA radiation (500 or 1000 mJ/cm^2^) or PDT (MAL 1mM plus 11.2 J/cm^2^ of red light) by fluorescence microscopy and flow cytometry (Figure 4).

A stronger signal of basal fluorescein in *GF* and *XF* compared with *CF* was observed (Figure 4A). Moreover, the signal was stronger after UVA exposure in all fibroblasts types but more clearly in *GF* and *XF*. Measurement of the mean fluorescence of fluorescein by flow cytometry (Figure 4B) confirmed the above findings: profiles showed that basal fluorescence was higher in *GF* and *XF* than in *CF*. UVA light induced an increase of fluorescence in all cell types compared to basal levels and it was significantly higher in *GF* and *XF* than in *CF* (*P*<0.05).

PDT also induced an increase in the fluorescence intensity of fluorescein in all cell types compared with baseline levels. Fluorescence values after treatment were significantly higher in *GF* and *XF* than in *CF* (*P*<0.05) (Figure 4C).

Overall, our results indicate that basal intracellular concentration of ROS is higher in *GF* and *XF* than in healthy fibroblasts. Moreover, both UVA light and PDT induce a higher oxidative response in *GF* and *XF* than in *CF*.

### Effects of MAL-PDT in UV-photoactivated fibroblasts

In order to assess whether PDT is effective under the effects of a mild exposure to UVR, we decided to stimulate fibroblasts with sub-genotoxic UV doses before applying PDT. We selected irradiation doses of 500 and 32 mJ/cm^2^ of UVA and UVB, respectively, and MAL-PDT treatment conditions of 1mM of MAL and 11.2 J/cm^2^ of red light, assuming that these conditions would allow an optimal activation of fibroblasts and a selective effect of PDT.

**Figure 5:**
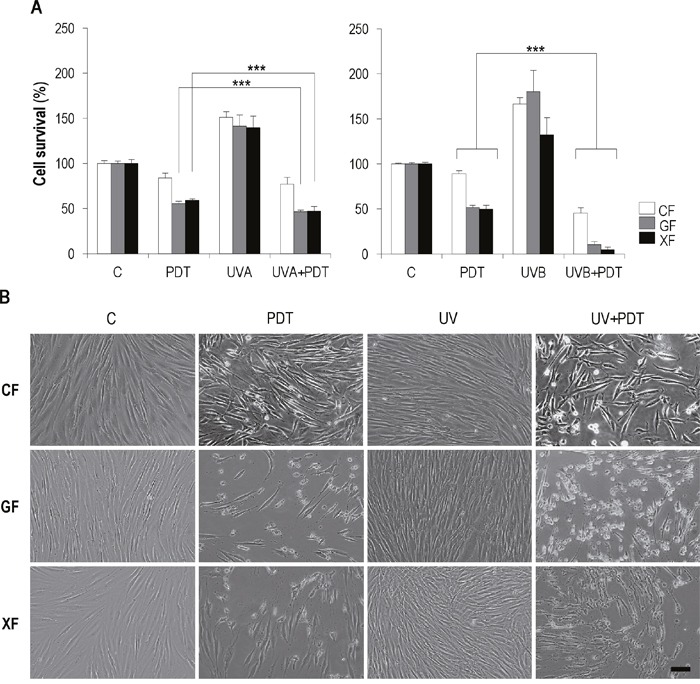
Effect of UV+PDT on fibroblasts survival and morphology Fibroblasts were subjected to UV light (500 mJ/cm^2^ of UVA or 32 mJ/cm^2^ of UVB) or UV+PDT treatments (500 mJ/cm^2^ of UVA or 32 mJ/cm^2^ of UVB followed by MAL 1mM plus 11.2 J/cm^2^). **(A)** Cell survival after treatments was quantified by trypan blue dye exclusion assay and counted using and Automated Cell Counter (only statistical differences between PDT and UV+PDT groups are shown; *P*<0.01). **(B)** Post-treatment cell morphology was analyzed by microscopic observation (Olympus BX-61) (scale bar: 40 μm).

In general, after UV+PDT, the cell death rate was higher than after PDT alone. In the case of UVA+PDT, compared to PDT, the decrease of survival was only significant in *GF* and *XF* cells (*P*<0.01), while *CF* showed a low sensitivity. When PDT was applied after stimulation with UVB light, the decrease of survival was significantly higher than that achieved with PDT alone for all fibroblast types (*P*<0.01), though the survival rate was significantly lower in *GF* and *XF* than in *CF* (*P*<0.01). Noteworthy, *XF* cells were the most sensitive to UVB+PDT, with a survival rate below 10% (Figure 5A).

Although more acute, morphological changes observed after UV+PDT (Figure 5B) were the same to those described after PDT alone for all the cell types: cytoplasmic retraction, cellular stretching and detachment of cells from substrate.

### DNA damage after PDT and UV+PDT

**Figure 6:**
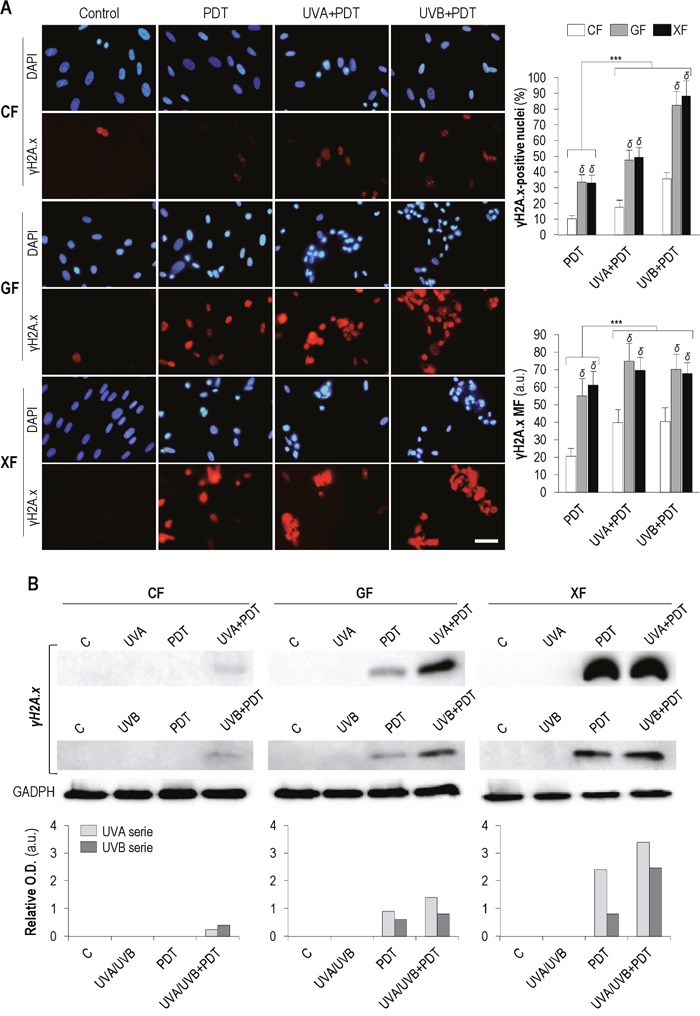
γH2A.x expression after PDT and UV+PDT Cell cultures were subjected to UV (500 mJ/cm^2^ of UVA or 32 mJ/cm^2^ of UVB), PDT (MAL 1mM plus 11.2 J/cm^2^) or UV+PDT treatments. **(A)** γH2A.x was detected by immunofluorescence 48 h after treatments. Its expression was observed by fluorescence microscopy and the percentage of γH2A.x-positive nuclei and the signal intensity of the protein were measured using the ImageJ software (*P*<0.01) (scale bar: 30 μm) (***: significant *P*<0.01; δ: significant differences with respect to *CF*, *P*<0.01) (MF: Mean Fluorescence). **(B)** Post-treatment expression of γH2A.x was also quantified by Western blot (Loading control: GADPH).

The expression of γH2A.x was analyzed 48 h after treatments by immunofluorescence (Figure 6A) and Western blot (Figure 6B). As shown Figure 6A, PDT and UV+PDT treatments lead to an increase in the percentage of γH2A.x-positive cells and in the signal intensity of this protein as compared to non-treated (control) fibroblasts (in which γH2A.x was only detected in dividing cells). When cells were subjected to UVA/UVB+PDT the amount of positive nuclei and the mean fluorescence were significantly increased with respect to PDT (*P*<0.01) in all fibroblast types. Moreover, percentage of positive cells and γH2A.x mean fluorescence after PDT or UV+PDT were significantly higher in *GF* and *XF* than in *CF* (*P*<0.01).

The results obtained by Western blot (Figure 6B) showed a similar trend. No expression of γH2A.x was observed in any of the fibroblasts types after UVA/UVB irradiation. In the case of *CF*, it was only detected after UV+PDT treatments, while in *GF* and *XF* both PDT and UV+PDT induced an increase in protein expression. Moreover, this increase was higher after UV+PDT than after PDT alone.

### Cell death mechanisms involved in PDT alone and after UV exposure

**Figure 7:**
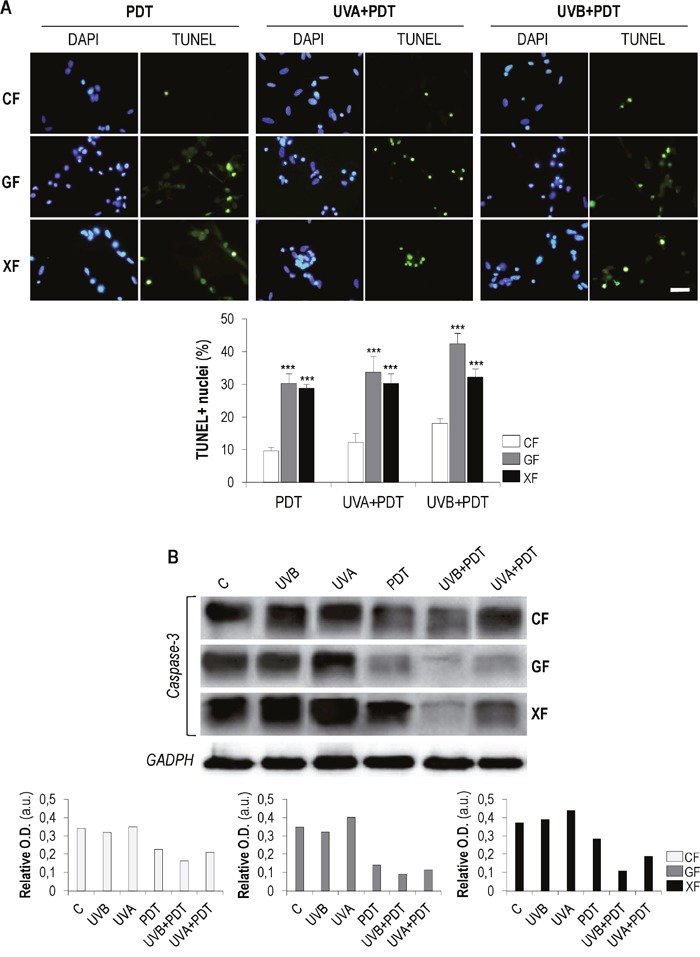
TUNEL assay and analysis of caspase-3 Fibroblasts were subjected to PDT (1 mM of MAL plus 11.2 J/cm^2^ of red light) or UVA/UVB irradiation (500 and 32 mJ/cm^2^, respectively) plus PDT. **(A)** TUNEL assay was performed 24 h after treatments.DNA fragmentation (green-stained fragments) typical of apoptotic processes was observed by fluorescence microscopy and the percentages of TUNEL-positive nuclei were measured using the ImageJ software (*P*<0.01) (scale bar: 20 μm). **(B)** Post-treatment expression of caspase-3 was quantified by Western blot.Loading control: GADPH (36 kDa).

We evaluated if apoptosis was the main cell death mechanism triggered by PDT alone and after UVA/UVB irradiation. We first performed a TUNEL assay 24 h after treatments to confirm if DNA fragmentation was occurring (Figure 7A). TUNEL assay detected the presence of DNA strand breaks in all PDT and UVA/UVB+PDT treated cultures. The number of TUNEL-positive nuclei was significantly higher in *GF* and *XF* than in *CF* (*P*<0.01). In addition, with the exception of *XF*, the percentage of TUNEL-positive cells was significantly higher in the case of UVA/UVB+PDT, as compared with PDT alone.

To further analyze the putative apoptotic cell death mechanism after treatments, we also evaluated the expression of caspase 3, a key mediator of this process, by western blot. In general, the expression of the inactive form of this protein (Figure 7B) showed a tendency to decrease when cells were subjected to PDT and that was more evident in cells subjected to UVA/UVB+PDT. This decrease was particularly remarkable after UVB+PDT. UVA and UVB irradiation alone did not induce any relevant difference in the expression of caspase 3, as compared to control cells. It can be expected that the decrease of the inactive form of caspase 3 was linked to an increase of its active forms, generated by the cleavage of the full-length protein.

One of the main cellular targets of active caspase 3 is PARP (Poly ADP Ribose Polymerase), mainly involved in DNA repair [24]. We therefore analyzed the expression levels of PARP after UVA/UVB, PDT and UVA/UVB+PDT by Western blot (Supplementary Figure 3). In the case of *CF*, only the active form of the PARP (116 kDa) was detected, and its expression decreased after PDT or UVA/UVB+PDT. Conversely, the inactive (cleaved) form of PARP (89 kDa) was observed in *GF* and *XF* treated with PDT or UVA/UVB+PDT.

## DISCUSSION

Minimally invasive treatments for skin cancers or pre-malignant lesions are highly desirable and PDT fulfills that modality. It is thus clinically relevant to achieve differential conditions enabling the elimination of cancer-prone cells while avoiding harmful effects on the unaffected tissue. Abundant evidence suggests that activated stromal fibroblasts play a major role in cancer development. Fibroblasts in the tumor stroma acquire a perpetually activated phenotype and become a subpopulation that can be identified by determined markers, although due to CAF heterogeneity there is a lack of universal markers [25–27]. Although malignancy is mainly associated to altered keratinocytes (i.e. in non-melanoma skin cancer), fibroblasts may be key players in the pathogenesis of cancer-prone genodermatoses such as XP and GS [19]. We have confirmed the cancer-associated fibroblasts (CAFs) features are present in fibroblasts obtained from XP and GS patients.

Alpha smooth muscle actin (α-sma), a general myofibroblast marker, is widely used to identify CAFs since they become reprogrammed variants resembling myofibroblasts [25–27]. Several studies have reported the increased expression of α-SMA in tumor surrounding tissue areas of facial BCCs [28] and nasopharyngeal carcinoma [27]. We showed here the overexpression of α-sma in *GF* and *XF* as compared with *CF*.

CAFs increase its migratory potential compared with normal fibroblasts [25, 29]. Cell migration is coordinated by a complex of proteins that localizes to sites of cell-matrix interaction, the focal adhesions (FAs). Vinculin plays a role in the regulation of these complexes and therefore this protein has also been described as a CAF marker. Our results show a higher expression of vinculin in *GF* than in *CF*.

Mesenchymal adhesion proteins, such as vimentin, are upregulated in cells with migratory properties. For that reason, vimentin has been also used as a marker to detect CAFs [22, 30]. In the present study, although no differences were found regarding to the signal intensity of this protein, vimentin filaments seemed better organized in *GF* than in *CF*.

Endoglin (CD105), a cell-surface glycoprotein identified as an optimal indicator of proliferation of human endothelial cells [31], was also evaluated here. Results showed that this protein was reduced in *GF* and *XF* as compared to *CF*. This novel finding is consistent with results described by Guerrero-Esteo et al. [32], which indicated that overexpression of endoglin in fibroblasts leads to decreased migration and invasion potential. Moreover, this was also reported by Liu et al. [33], who indicated that loss of endoglin expression appears to be associated with prostate cancer progression.

Taken together, all these results provide support to consider that primary fibroblasts obtained from GS and XP patients could be potential CAFs. In accordance to that, a previous study carried out by Gache et al. [19] in human fibroblasts obtained from skin biopsies of nevoid basal cell carcinoma syndrome (NBCCS) patients suggested that susceptibility of these patients toward BCCs development could be partly due to a “pre activated state” of stromal fibroblasts.

Our results showed a selectivity of MAL-PDT towards dermal fibroblasts from XP and GS patients. Administration of MAL at 1mM followed by 11.2 J/cm^2^ of red light leads to a differential effect on fibroblasts viability: survival of *GF* and *XF* decreased by almost 40% while in *CF* only diminished a 5%. Several clinical trials carried out in Gorlin syndrome patients describe PDT as an interesting treatment option. Application of topical ALA or MAL-PDT in multiple BCCs not only reduces the thickness of lesions but also achieves clearance rates between 60-80%, with excellent cosmetic outcomes [34–36]. Moreover, systemic PDT using Photofrin as photosensitizer and interstitial optical fibers as irradiation source, results effective in the treatment of thicker BCCs (as nodular subtypes), avoiding surgery [11, 12]. In addition, Caty et al. [37], reported that MAL-PDT is able to delay de development of BCCs in PTCH heterozygous mice model chronically exposed to UV radiation, which suggest the preventive role of PDT in this type of lesions. In contrast to GS, there is little evidence in the literature supporting the use of PDT in XP patients. Larson and Cunningham [17] reported a case of XPC patient treated with ALA-PDT and blue light without adverse events. And a study performed by Segura et al. (2011) [18], concluded that MAL-PDT may be useful for the treatment of superficial BCCs in XP patients, with minimal secondary effects.

Differential impact of PDT described above is supported by measurements of intracellular ROS concentration: ROS production triggered by PDT is higher in fibroblasts from GS and XP patients.

We also checked if effectiveness and selectivity observed after MAL- PDT were maintained when cells were exposed to UVA, a dermis-acting radiation, and UVB under mild irradiation conditions. To assess for potential unexpected DNA/chromatin damage of UVR doses we analyzed γ-H2AX. This protein is a minor component of nuclear histone H2A involved in several biological processes, including specific aspects of cell division (structural and functional chromatin regulation during mitosis), stem cell biology and aging [38]. The phosphorylation of this histone was originally identified as an early event after the direct formation of DNA double-strand breaks (DSBs) by ionizing radiation. However, the generation of γ-H2AX is now also considered to occur during DNA repair process at sites of various types of DNA damage, such as UV-induced photolesions [39, 40]. Our results with this marker confirmed that UVA/UVB doses used to activate *CF*, *GF* and *XF*, do not induce DNA damage. As pointed out above, γH2A.x is normally involved in cell division process, so the observed expression in dividing cells cannot be attributed to UV-induced damage.

In addition, formation of CDPs is cause of UV-induced cytotoxicity and mutagenicity, playing an important role in photocarcinogenesis [41]. In this work, CPDs evaluation confirmed that the low doses of UVA/UVB used to stimulate fibroblasts did not induce detectable damage.

Exposition to UV light and subsequent application of PDT to treat primary fibroblasts and keratinocytes from GS and XP patients has not been described. Here we show that GS and XP patient-derived fibroblasts (potentially CAFs) are more sensitive to PDT than fibroblasts from healthy donor not only at basal conditions but also when are stimulated by UVA and UVB light.

It is widely documented the involvement of caspase-3 in apoptosis, being a critical executor of this death process. Its activation take place by the proteolytic processing of its inactive zymogen into activated p17 and p12 fragments [42]. On the other hand, PARP plays a crucial role in DNA repair and is one of the most important targets of caspase-3. The cleavage of PARP by caspase-3 inactivates it and inhibits PARP's DNA-repairing abilities [43]. Taking into account our results of TUNEL assay, it seems that the three types of fibroblasts undergo apoptosis when treated with PDT or UV+PDT. Post-treatment analysis of caspase-3 and PARP expression pointed out that apoptosis is the main mechanism of cell death. The decrease of inactive caspase-3, accompanied by increased expression of cleaved PARP, supports that PDT and UV+PDT could be inducing programmed fibroblasts death. Nevertheless, percentages of apoptotic figures found in treated cultures determined by TUNEL assay were lower than overall death percentages resulting from viability assays, so other cell death pathways could be also be activated.

Overall our results show susceptibility of GS and XP fibroblasts to PDT. Such therapeutical approach evaluated here in cells from genetic diseases, could be also beneficial for spontaneous skin tumors where CAFs play a significant role. The fact that these fibroblasts behave as CAFs is likely contributing to malignancy in the genodermatoses addressed in our study and, since they are highly susceptible to MAL-PDT, suggest that this approach could not only be relevant to treat the epithelial component of tumors or premalignant lesions but also the activated stromal cells. As shown in our study, a priming response elicited by UVA (the one with the capacity to reach the dermis) may even enhance the effect of MAL-PDT.

## MATERIALS AND METHODS

### Cell culture

Human dermal fibroblasts were isolated from XP (Xeroderma Fibroblasts, *XF*) and GS (Gorlin Fibroblasts, *GF*) patients. Fibroblasts obtained from three different healthy donors were used as control (Control Fibroblasts, *CF*). Isolation was performed according to current procedures [44, 45]. Fibroblasts were cultured up to eight passage under standard conditions in Dulbecco's modified Eagle's medium (DMEM) (Thermo Fisher Scientific Inc., Walthan MA USA).

### Immunofluorescence

For immunofluorescence, cells were grown on glass coverslips, fixed with 3.7% formaldehyde in PBS and permeabilized in 0.1% Triton X-100 in PBS (Merck, Darmstadt, Germany). Then, cells were incubated with the primary antibodies for 1 h, washed with PBS and incubated with secondary antibodies for 45 min. At last, cells were mounted with ProLong Gold antifade with DAPI (Thermo Fisher Scientific Inc., Walthan MA USA). In the case of CPDs detection, cells were also treated with 2M HCl for DNA denaturation before incubation with the corresponding primary antibody. Antibodies used for immunofluorescence are shown in the Supplementary Table 1.

### UV irradiation, photodynamic therapy and UV+PDT treatments

When fibroblasts, seeded in 35 mm dishes (Thermo Scientific Inc., Walthan MA USA), reached a confluence of 30-40%, were washed with PBS and exposed to UVB/UVA light under a thin film of PBS. Lamps were maintained at 18 cm from cultures. UVB doses applied were 21, 32 and 64 mJ/cm^2^ and UVA doses were 500 and 1000 mJ/cm^2^. Immediately after irradiation, PBS was replaced with fresh culture medium. The UVA source was a 50/60 Hz 14 VA lamp (300-400 nm; Camag Scientific, Muttenz, Switzerland) (Supplementary Figure 1A). The UVB source was a 2×15 W-312 nm tube UV lamp (270-380 nm, Vilber Lourmat, Marne La Vallée, France) filtered through a colored-glass filter with a 305± 5 nm cut-on wavelength (Newport, Irvine, CA, USA) (Supplementary Figure 1B). UVB and UVA spectra and outputs were measured using a USB2000+ radiometer (Ocean Optics, Dunedin, Florida, USA).

For PDT administration, fibroblasts were incubated in serum-free medium containing methyl-δ-aminolevulinic acid (MAL, Sigma Aldrich, St. Louis, MO, USA), a precursor of the endogenous photosensitizer protoporphyrin IX (PpIX), at a final concentration of 1 mM, for 5 h and in the dark (MAL concentration and incubation period were previously tested and selected as optimal treatment conditions). Then, medium was replaced by fresh medium with 10% FBS and the cells were irradiated with different doses of red light, using a 384 light-emitting diode matrix (WP7143 SURC/E Kingsbright, wavelength 636±17 nm).

To perform the UVB/UVA+PDT treatments, cells were first exposed to UVB/UVA light and, 48 h after irradiation, subjected to MAL-PDT.

### Determination of cell survival/proliferation

Cell survival was assessed by the trypan blue dye exclusion assay. At different time points after treatments adherent cells were trypsinized, collected with floating cells and pelleted by centrifugation (1200 rpm, 5 min). Then, cells were re-suspended in PBS mixed with 0,4% trypan blue solution (Sigma Aldrich, St. Louis, MO, USA) and counted by using an Automated Cell Counter TC20^TM^ (BioRad, Hercules, CA, USA).

### Measurement of intracellular ROS

In order to analyze the intracellular production of ROS after UVA irradiation or PDT, dihydrofluorescein diacetate (DHF-DA) assay was performed. This is a fluorescent probe for detecting intracellular oxidants because of its high reactivity toward specific oxidizing and one of the most common methods to detect intracellular ROS levels species. After incorporating into the cell, DHF-DA is deacetylated by cellular esterases to a non-fluorescent compound, which is later oxidized by ROS giving fluorescein, a highly fluorescent compound, which can be detected by fluorescence microscopy or spectroscopy with maximum excitation and emission spectra of 495 nm and 529 nm, respectively [46]. A stock 0.5 mM of DHF-DA (Abcam) in absolute ethanol was prepared. Work solution for cell incubation was a dilution from this stock in DMEM without FBS to a final concentration of 6 μM. For detecting ROS after UVA irradiation, fibroblasts were seeded in 100 mm culture dishes and maintained in the incubator until they reached a confluence of 80-90%. Then, cells were incubated for 50 min with 6 μM of DHF-DA. At the end of the incubation period and without removing DHF-DA, cells were exposed to UVA light (500 or 1000 mJ/cm^2^). Fibroblasts incubated with DHF-DA but not exposed to UV radiation were used as DHF-DA control. Immediately after irradiation, cells were analyzed by fluorescence microscopy, under blue excitation light, or were washed twice with PBS, trypsinized with Trypsin-EDTA 0.05% and centrifuged for 10 min at 2000 r.p.m. The pellet was fixed for 15 min in 3.7% formaldehyde/PBS solution, centrifuged for 5 min at 2000 r.p.m. and resuspended in PBS. Fluorescence of fluorescein was measured by flow cytometry (Cytomics 4500). For detection of ROS after PDT, cells were seeded in 25 cm^2^ culture flasks and incubated with 1mM of MAL for 5 h in the dark. DHF-DA 6 μM was administered in the last 50 min and finally cells were exposed to 11.2 J/cm^2^ of red light. After irradiation, cells were processed and fluorescein fluorescence was measured as described above.

### DNA fragmentation assay

Terminal deoxynucleotidyl transferase dUTP nick end labeling (TUNEL) assay was used to determine DNA fragmentation. At specific points after treatments, cells were washed with PBS, fixed with 3.7% formaldehyde and permeabilized in 0.1% Triton X-100/PBS. DNA ends were labeled with an *in situ* cell death detection kit (Roche, Risch-Rotkreuz, Switzerland) for 1 h at 37°C and the samples were mounted as for immunofluorescence.

### Western blot

Twenty-four hours after treatments, cells were lysed with RIPA buffer (BioWorld, Dublin, OH, USA) containing phosphatase and protease inhibitor cocktails (Roche, Risch-Rotkreuz, Switzerland). The samples were adjusted to the same protein concentration (BCA protein assay kit, Pierce, Rockford, IL, USA) and denatured by boiling in Laemmli sample buffer with 5% β-mercaptoethanol. Then were subjected to electrophoresis separation in SDS-PAGE. Gels were transferred to a PVDF membrane using a Trans-Blot turbo transfer system (BioRad, Hercules, CA, USA) and membranes were blocked with 5% nonfat milk in Tris-buffered saline (TBS) with 0.1% Tween 20, for 2 h. After blocking, membranes were incubated overnight with specific primary antibodies and then with HRP-conjugated secondary antibodies (Supplementary Table 1) and developed by chemiluminiscence (ECL, Thermo Fisher Scientific Inc., Walthan MA USA) using a ChemiDoc system (BioRad, Hercules, CA, USA). Bands corresponding to the different proteins were quantified, digitalized and analyzed with Image Lab 2.0.1. software (BioRad, Hercules, CA, USA) and Adobe PhotoShop CS5 12.0 (Adobe Systems Inc., USA).

### Microscopic observations, quantification and statistical analysis

Microscopic observation was realized using a fluorescence microscope (Olympus BX-61) equipped with the following filter sets: ultraviolet (UV, 365 nm, exciting filter UG-1), blue (450−490 nm, exciting filter BP 490), green (545 nm, exciting filter BP 545) and red (620-700 nm, exciting filter CC50R). Images were obtained with the digital camera Olympus CCD DP70 and processed using the Adobe PhotoShop. To quantify the expression of CAF markers, mean fluorescence signal intensity of each marker in a total area of 4 cm^2^ was measured. Measurement was performed from microscopy images, using the Measure and Label plugin of ImageJ 1,43u (NIH, USA). In the case of CPDs, γ-H2AX and TUNEL assay, positive nuclei for these markers were counted (from a total of 800 nuclei of each treatment condition) from microscopy images using the Cell Counter plugin of ImageJ 1.50b (NIH, USA). Moreover, mean fluorescence *per* nucleus was quantified too through ImageJ.All experiments were carried out using three replicates in three independent experiments. The results were processed using software SPSS Statistics 20.0 (IBM^®^). Data are expressed as the mean value ± standard deviations (SD). The statistical significance was determined using the analysis of variance (ANOVA) and t test (*: *P*<0.1; **: *P*<0.05; ***: *P*<0.01).

## SUPPLEMENTARY MATERIALS FIGURES AND TABLES



## Abbreviations

UVA: (ultraviolet light A)
UVB: (ultraviolet light B)
PDT: (photodynamic therapy)
MAL: (methyl δ-aminolevulinic acid)
PS: (photosensitizer)
CAF: (cancer-associated fibroblast)
XP: (xeroderma pigmentosum)
GS: (Gorlin-Goltz syndrome)
CPD: (cyclobutane pyrimidine dimers)
ROS: (reactive oxygen species)
