# Supplementary material for: Effects of photodynamic therapy on dermal fibroblasts from xeroderma pigmentosum and Gorlin-Goltz syndrome patients
Source: Oncotarget. 2017 Aug 24;8(44):77385–99. doi: 10.18632/oncotarget.20485 (PMC5652786; doi:10.18632/oncotarget.20485)
Supplement: Supplementary file 1 [file oncotarget-08-77385-s001.pdf]

Effects of photodynamic therapy on dermal fibroblasts from xeroderma pigmentosum and Gorlin-Goltz syndrome patients

SUPPLEMENTARY MATERIALS

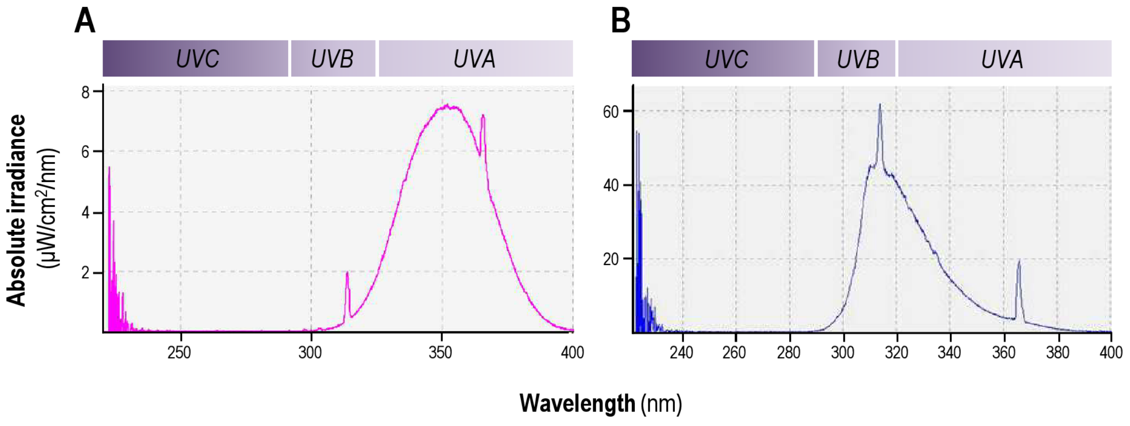

Supplementary Figure 1: Spectra of UV light sources. Power and wavelength range of UVA **(A)** and UVB **(B)** sources, measured using the USB2000+ radiometer (Ocean Optics, Dunedin, Florida, USA).

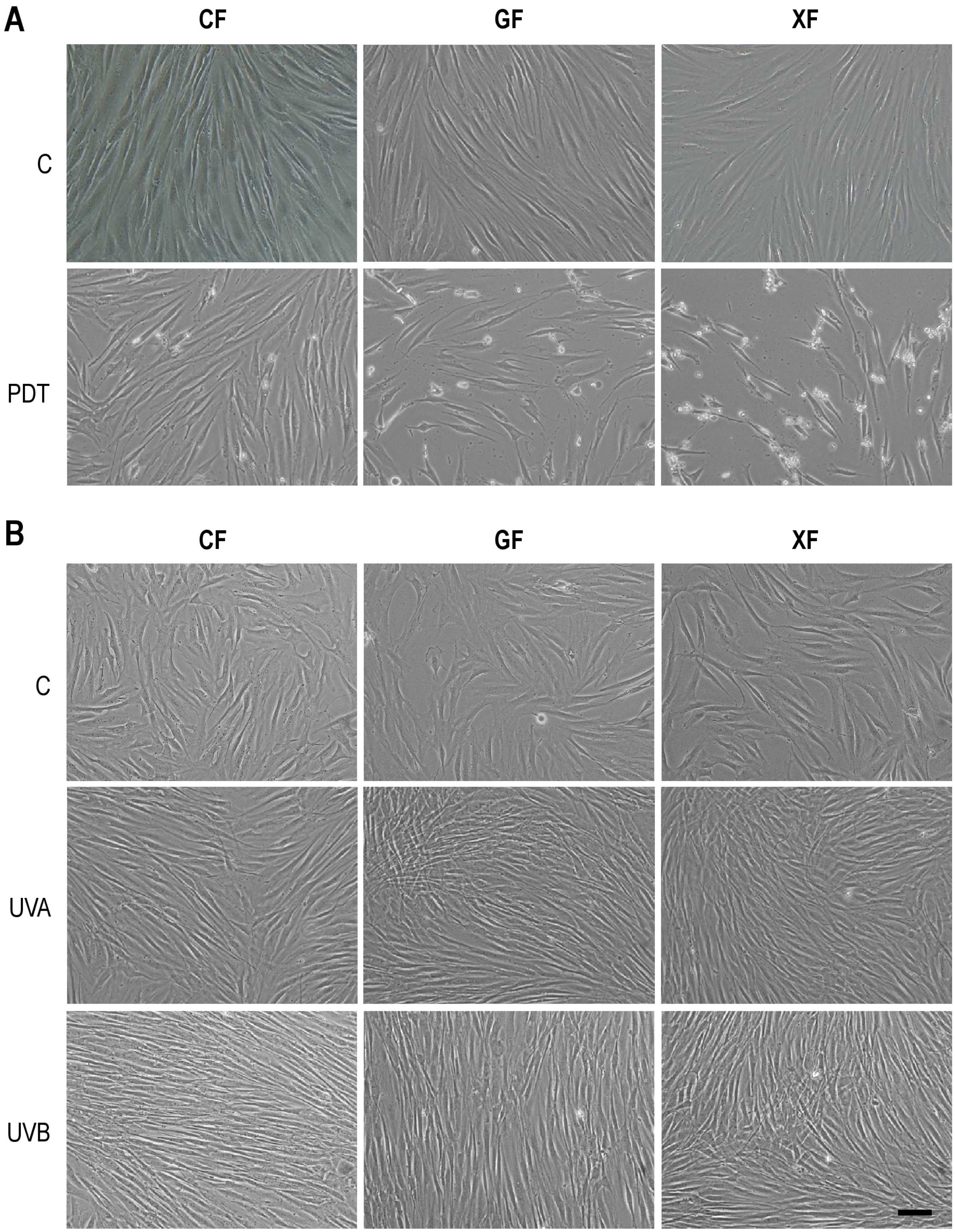

Supplementary Figure 2: Effects of MAL-PDT and UV irradiation on fibroblasts morphology. (A) The contrast phase images correspond to cell cultures 24 h after PDT (MAL 1mM plus 11.2 J/cm^2 of red light). **(B)** *CF*, *GF* and *XF* cultures were subjected to UVA (500 mJ/cm^2) or UVB (32 mJ/cm^2) light. Morphology was analyzed by microscopic observation. (scale bar: 80 μm).

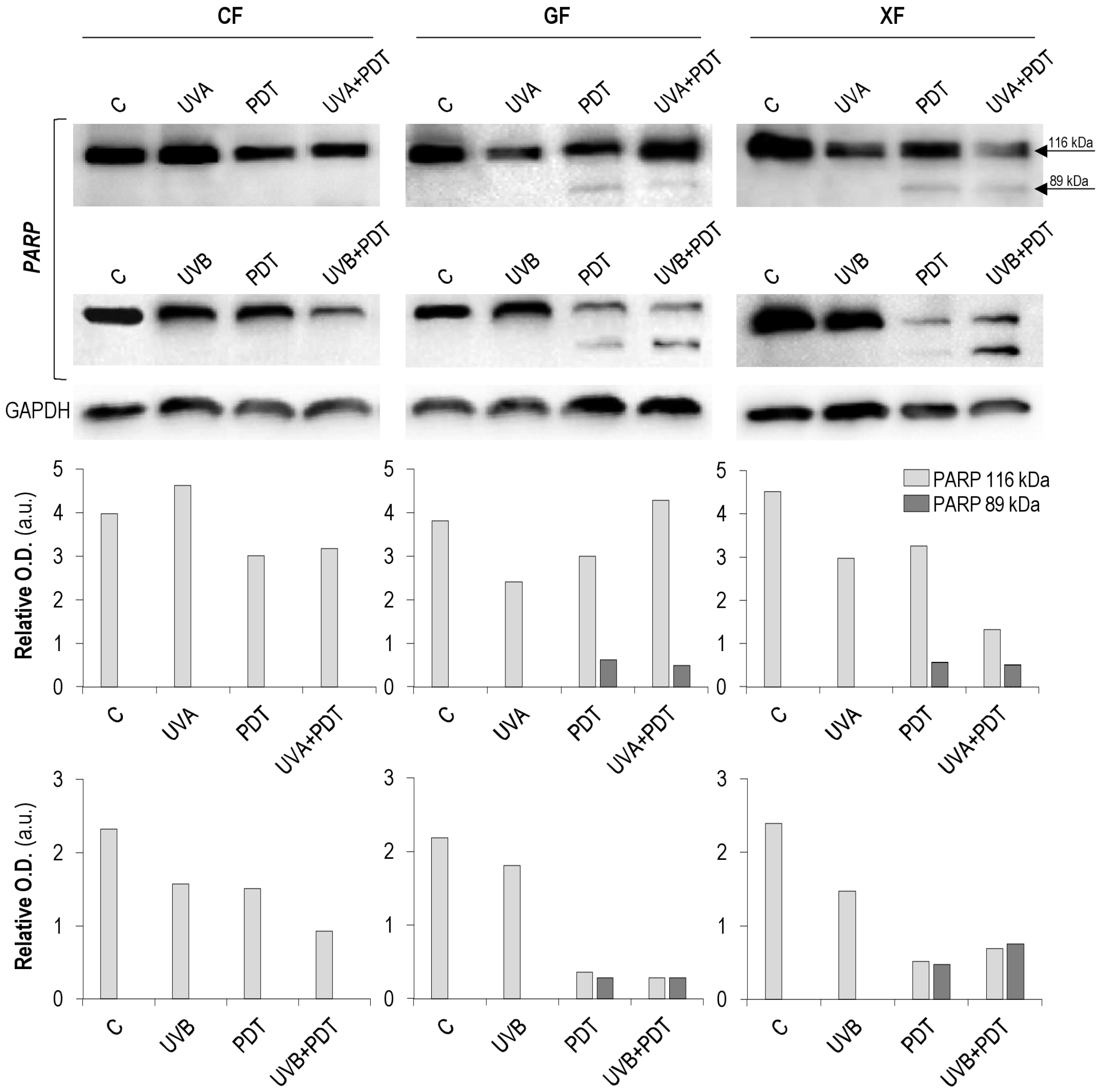

Supplementary Figure 3: Effect of UV, PDT and UV+PDT treatments on PARP expression. Cell cultureswere subjected to UV (500 mJ/cm^2 of UVA or 32 mJ/cm^2 of UVB), PDT (MAL 1mM plus 11.2 J/cm^2) or UV+PDT treatments. Expression of both total and inactive (cleaved) form of PARP (116 and 89 kDa, respectively) was evaluated by Western blot. Loading control: GADPH (36 kDa).

Supplementary Table 1: Antibodies used for immunofluorescence (IF) and Western blot (WB)

Antigen	Origin	Source	Application	Assay
α-smooth muscle actin (α-sma)	Rabbit (polyclonal)	Cell signaling	IF	CAF marker
Vinculin	Mouse (monoclonal)	Sigma	IF	CAF marker
Vimentin	Rabbit (polyclonal)	Abcam	IF	CAF marker
Endoglin	Mouse (monoclonal)	Dako	IF	CAF marker
cyclobutane pyrimidine dimer (CPD) (clone TDM2)	Mouse (monoclonal)	Cosmo Bio Co Ltd.	IF	DNA damage
γ-H2AX (phophorylated at Ser139)	Rabbit (polyclonal)	Cell signaling	IF/WB	DNA damage
caspase-3	Rabbit (polyclonal)	Cell signaling	WB	Cell death
poly ADP-ribose polymerase (PARP)	Rabbit (polyclonal)	Cell signaling	WB	Cell death
glyceraldehyde 3-phosphate dehydrogenase (GADPH)	Rabbit (polyclonal)	Cell signaling	WB	Loading control
mouse IgG -Alexa 488	Goat (polyclonal)	Molecular probes	IF	Secondary antibodies
rabbit IgG -Alexa 546	Goat (monoclonal)	Molecular probes	IF	
mouse IgG-HRP conjugated	Goat (polyclonal)	Fisher Scientific	WB	
rabbit IgG-HRP conjugated	Goat (polyclonal)	Fisher Scientific	WB	

Antigen, origin, source and application of primary and secondary antibodies used to perform immunofluorescence and Western blot assays.
